# Asymmetrical nature of the *Trollius*–*Chiastocheta* interaction: insights into the evolution of nursery pollination systems

**DOI:** 10.1002/ece3.1544

**Published:** 2015-10-08

**Authors:** Tomasz Suchan, Mélanie Beauverd, Naïké Trim, Nadir Alvarez

**Affiliations:** ^1^ Department of Ecology and EvolutionUniversity of LausanneBiophore Building1015LausanneSwitzerland; ^2^ Institute of Environmental BiologyUniversity of Wrocławul. Kanonia 6/850‐328WrocławPoland

**Keywords:** Asymmetrical interactions, brood‐site pollination, *Chiastocheta*, conditional outcomes, mutualism, plant–pollinator interactions, pollinator community, pollinator loss, reproductive ecology, reproductive success, *Trollius europaeus*

## Abstract

The mutualistic versus antagonistic nature of an interaction is defined by costs and benefits of each partner, which may vary depending on the environment. Contrasting with this dynamic view, several pollination interactions are considered as strictly obligate and mutualistic. Here, we focus on the interaction between *Trollius europaeus* and *Chiastocheta* flies, considered as a specialized and obligate nursery pollination system – the flies are thought to be exclusive pollinators of the plant and their larvae develop only in *T. europaeus* fruits. In this system, features such as the globelike flower shape are claimed to have evolved in a coevolutionary context. We examine the specificity of this pollination system and measure traits related to offspring fitness in isolated *T. europaeus* populations, in some of which *Chiastocheta* flies have gone extinct. We hypothesize that if this interaction is specific and obligate, the plant should experience dramatic drop in its relative fitness in the absence of *Chiastocheta*. Contrasting with this hypothesis, *T. europaeus* populations without flies demonstrate a similar relative fitness to those with the flies present, contradicting the putative obligatory nature of this pollination system. It also agrees with our observation that many other insects also visit and carry pollen among *T. europaeus* flowers. We propose that the interaction could have evolved through maximization of by‐product benefits of the *Chiastocheta* visits, through the male flower function, and selection on floral traits by the most effective pollinator. We argue this mechanism is also central in the evolution of other nursery pollination systems.

## Introduction

*Ethically, there is nothing in the phenomena of symbiosis to justify the sentimentalism they have excited in certain writers. Practically, in some instances, symbiosis seems to result in mutual advantage. In all cases it results advantageously to one of the parties, and we can never be sure that the other would not have been nearly as well off, if left to itself*. Roscoe Pound (1983)

Mutualisms are often viewed as one‐to‐one interactions, obligate and unconditionally beneficial for both partners (Stanton 2003). However, such textbook definition does not reflect all the diversity observed in nature. In fact, one‐to‐one mutualisms seem to be extremely rare (Hoeksema and Bruna 2000), and focusing on them reflects more the human preference for illustrative examples, than their prevalence (Bronstein et al. 2006). Indeed, most mutualisms include whole assemblages of interacting species. For instance, pollination mutualisms are more generalist and variable than previously thought (Ollerton 1996; Waser et al. 1996; Ollerton et al. 2009; but see Johnson and Steiner 2000; Fenster et al. 2004), and even in apparently specific pairwise systems, closer examination usually reveals guilds of interacting partners (Cook and Rasplus 2003). In such systems, the different partners do not appear showing the same level of specificity to each other, and several authors have acknowledged the asymmetrical (i.e., specialists tend to interact with more generalist species and *vice versa*) – rather than symmetrical – nature of plant–pollinator interactions (Bascompte et al. 2003; Vázquez and Aizen 2004). Yet, most studies seem to focus on pairwise interactions; however, even for the very specialized ones, analyzing the interaction without the biotic context, that is, other potential partner species, is unrealistic (Bronstein et al. 2003; Price et al. 2005).

In the case of nursery pollination systems, the pollinators lay eggs on the developing fruit and their larvae act as seed parasites (Sakai 2002; Dufaÿ and Anstett 2003). As such, their pollination service is costly for the plant (Bronstein 2001), and the interaction outcome may depend on the availability of other pollinators, providing pollination benefits at a lower cost, thus making the mutualism conditional (Bronstein 1994). This has been illustrated in systems characterized by lower levels of specialization, such as in *Silene*‐*Hadena*, in which the cost versus benefit for the plant changes depending on the community context (Pettersson 1991; Kephart et al. 2006; Reynolds et al. 2012). There, pollinators other than seed‐eating partners can provide pollination service, and the relative outcome of the interaction depends on their availability. In other systems, such as *Silene*‐*Delia* (Pettersson 1992) or *Lithophragma*‐*Greya* (Thompson and Pellmyr 1992), the abundance of other effective co‐pollinators swamps the possible mutualistic effects of the seed‐eating pollinator, completely switching the interaction outcomes to antagonistic.

In contrast, the influence of such external factors is much lower in systems showing high levels of specialization, such as the yucca–yucca moth (Addicott 1986; Pellmyr 1997), the senita cactus–senita moth (Fleming and Holland 1998), the fig–fig wasp (Janzen 1979), and leafflower–leafflower moth systems (Kato et al. 2003). Above‐mentioned systems are the most spectacular plant–insect mutualisms in general, with the insects actively transferring pollen to the stigma. The obligate nature of these interactions, defined by precise functional adaptations, generally excludes other pollinators, making the outcome of the interaction independent of the surrounding insect communities.

The evolutionary transition from the systems with variable outcomes to those featuring a high degree of specificity among partners is still weakly understood (Mayer et al. 2011). It is thus of prime importance to study intermediate systems to get an insight into processes and mechanisms of origin and maintenaince of mutualisms. Among the few that lie at the boundary between obligate and mutualistic interactions (as in figs or yucca), and facultative with variable outcomes (such as in *Lithophragma* and *Silene*), is the interaction between the European globeflower *Trollius europaeus* (Ranunculaceae) and anthomyiid flies within the genus *Chiastocheta* (Diptera: Anthomyiidae). So far, this system has been treated as a strict mutualism (Pellmyr 1989; Jaeger and Després 1998; Després et al. 2007). *Chiastocheta* flies passively pollinate the plant, but also lay eggs on the carpels, and the developing larvae feed on the seeds (Pellmyr 1989). Although the obligate nature of *Trollius*–*Chiastocheta* interaction has been postulated for a long time (Pellmyr 1989), it has never been tested in a direct way, that is, by measuring fitness in the populations where *Chiastocheta* are not present. Here, we examine processes occurring in plant populations having lost their pollinators and investigate changes in the interaction outcomes before and after this loss.

Based on previous works, claiming the obligatory nature of this mutualism, we hypothesize that plant fitness should dramatically decrease without *Chiastocheta*. The alternative hypothesis would be that if the interaction is not truly symmetrical, that is, obligate only for the flies (Bascompte et al. 2003; Vázquez and Aizen 2004), we should expect non‐negligible seed production. If the plant is able to reproduce when *Chiastocheta* are absent, the antagonistic *versus* mutualistic outcome of the interaction can be assessed by comparing the number of seeds produced, and their germination, between the populations where the flies are present and absent.

To verify above hypotheses, we took the opportunity of a natural experiment. We monitored the seed set and selected fitness‐related traits in the progeny (i.e., seed mass and germination rate) in several highly isolated *T. europaeus* populations, in some of which *Chiastocheta* communities went extinct. This allowed us to directly test the hypothesis of the obligate nature of the interaction from the plant's perspective. Moreover, by monitoring several populations over consecutive years, we could disentangle the effect of *Chiastocheta* presence on the fitness from the site‐specific effects.

## Materials and Methods

### Study system and study area

The European globeflower *T. europaeus* L. (Ranunculaceae) is a perennial arctic–alpine plant distributed throughout Northern, Central, and in the mountains of Southern Europe (Hultén and Fries 1986). It produces hermaphroditic and homogamous yellow flowers, reaching up to 5 cm in diameter. Each flower consists of about 30 carpels with approximately 12 ovules, surrounded by many stamens, 5–15 nectariferous staminodia and sepals that form a yellow globe around the reproductive structures. The plant is 10‐ to 70‐cm high and often grows in dense tussocks. Each of the stalks has one to three, rarely more, flowers (Tutin et al. 1964; Doroszewska 1974). The European globeflower is self‐incompatible; however, a very small degree of selfing was observed in natural populations (Pellmyr 1989; Jaeger and Després 1998; Lemke and Porembski 2012). Flowering occurs almost simultaneously within populations and usually lasts for 2–3 weeks, each flower lasting for around a week, or longer when the conditions are unfavorable, for example, due to long periods of rain (Pellmyr 1989; Jaeger and Després 1998). In the study area, flowering starts in early May.

Seven *Chiastocheta* species are known to occur in Europe: *Chiastocheta dentifera*,* Chiastocheta inermella*,* Chiastocheta lophota*,* Chiastocheta macropyga*,* Chiastocheta rotundiventris*,* Chiastocheta setifera,* and *Chiastocheta trollii*, all of which exclusively and obligatorily reproduce on the fruits of *T. europaeus* (Pellmyr 1989, 1992; Jaeger and Després 1998). During the flowering period, flies visit only *Trollius* flowers, and, similarly to previous studies (Ibanez et al. 2009), we did not observe *Chiastocheta* visiting any other plant species. During flower visits, flies passively pollinate flowers while they feed, mate, and oviposit. We also observed that *Chiastocheta* find shelter inside the flowers during unfavorable weather conditions, for example, during cold or rainy days. Females oviposit on carpels and larvae feed on developing seeds, with species‐specific mining patterns (Pellmyr 1989; Pompanon et al. 2006). After fruit dehiscence, larvae pupate in the ground and overwinter. We did not collect data on *Chiastocheta* community species composition from the studied localities, as we did not sample flies from such isolated populations for conservation reasons. Nonetheless, in the neighboring area of the Sudety Mountains, where *T. europaeus* populations are abundant, four morphospecies of *Chiastocheta* were recorded: *C. dentifera*,* C. inermella*,* C. rotundiventris,* and *C. trollii* (T. Suchan, unpubl. data).

**Figure 1:**
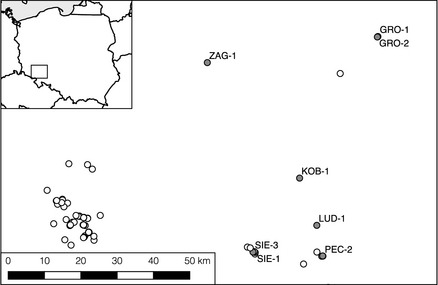
Location of the studied *Trollius europaeus* populations in Southeastern Poland (grey points) and other plant populations in the neighboring area (white points).

The study area is situated in Southwestern Poland, in Lower Silesia province, in the vicinity of Wrocław city (study area: 16°33′–17°13′E; 50°47′–51°20′N). The mean annual precipitation in the region lies between 566 and 590 mm. The mean temperatures vary from 7.1°C to 18.8°C in May and from 10.7°C to 22°C in June (annual mean 8.3°C). All the studied populations are situated between 119 and 207 m above sea level (Fig. 1; Table 1). In the study area, *T. europaeus* occures in a previously continuous range of the species (Hultén and Fries 1986), but experienced a rapid decline caused by agricultural intensification – a situation similar to eastern Germany (Lemke 2011). These remnant populations are highly isolated, occupying relics of tall‐herb, extensively mowed, moist meadows. Local extinctions of *Chiastocheta* communities have occurred in a substantial number of them due to disturbance events, such as grazing by herbivores or mowing. High isolation prevents quick recolonization of extinct sites, thus providing an opportunity to address questions on the interaction specificity.

**Table 1:** *Trollius europaeus* populations sampled for each year, with population size, information on *Chiastocheta* presence, and the number of fruits sampled

Population	Year	Number of flowers	*Chiastocheta*presence (P) or absence (A)	Fruits sampled
GRO‐1	2010	60	P	22
	2011	22	P	4
GRO‐2	2009	2700	P	40
	2010	2000	P	21
	2011	3000	P	39
KOB‐1	2009	1700	P	24
	2010	17300	A	157
	2011	14400	A	50
PEC‐2	2009	14400	P	50
	2010	5500	A	88
	2011	16900	A	35
LUD‐1	2009	600	P	33
SIE‐1	2009	13	P	8
SIE‐3	2011	8500	P	71
ZAG‐1	2011	5	A	5

### Populations sampled

For assessing the seed set, fruits of *T. europaeus* were collected in 2009, 2010, and 2011, in eight populations (Table 1). Fruits were randomly collected in each population 1–2 weeks after the end of flowering, between the end of May and the beginning of June. Care was taken to always sample one, top fruit per stem to avoid any putative effect of flower position (Hemborg and Després 1999). In addition, the fruits were collected in order not to repeat the sampling from one plant, to avoid pseudoreplication issues. Three populations were sampled over three consecutive years (GRO‐2, KOB‐1, and PEC‐2), one over 2 years (GRO‐1 in 2010 and 2011), and four during 1 year (LUD‐1 and SIE‐1 in 2009, SIE‐3, and ZAG‐1 in 2012). The reason that not all the populations could be sampled in three consecutive years was complete loss of fruits, either because of herbivory (GRO‐1, SIE‐1) or lack of flowering in the population resulting from early mowing (LUD‐1). In addition, two populations – SIE‐3 and ZAG‐1, were discovered in the third, final year of the study. All populations except LUD‐1 were not subject to agriculture (i.e., mowed or used as pastures) during the study period.

The presence or absence of *Chiastocheta* was determined by observing *T. europaeus* flowers in the studied populations upon several visits during the flowering period (T. Suchan, unpubl. data) and confirmed by checking the fruits for the presence of eggs. Two of the populations sampled for three consecutive years lost the pollinators before the second field season (KOB‐1 and PEC‐2), and additionally, we found a third population without *Chiastocheta* in the last year of the study (ZAG‐1). We therefore have sampled five population × year combinations without *Chiastocheta* and ten with the flies present.

The number of flowers present in each population was used as a proxy for the plant population size (Johannesen and Loeschcke 1996; Klank et al. 2010; Lemke and Porembski 2012). For small populations, that is, under 1000 flowers, we assessed the population size by direct counting of flowers. For larger populations, the mean population density was calculated by recording the number of flowers in a 2‐m‐wide transect and dividing it by the transect area. Then, the population size was extrapolated by multiplying the mean flower density by the population area. As we observed variability in the number of flowers produced in each population over the years, the population size per each year was used in the analyses.

### Seed set and seed predation

The seed set of the plant was defined as the ratio of developing seeds to ovules, evaluated before predation by *Chiastocheta* larvae occurred, but at the time when developing seeds could be distinguished from nondeveloping ovules or aborted seeds. Therefore, this parameter does not account for subsequent seed loss due to predation and is equivalent to the *seed initiation frequency* as defined by Pellmyr (1989), *seed set* as in Jaeger and Després (1998), or *relative seed set* as in Lemke and Porembski (2012). For each collected fruit, we counted the number of developing seeds in a random subsample of five carpels. It was previously shown by Pellmyr (1989) that assessing the seed set in a subsample of five carpels per fruit by dividing the number of developing seeds by the total number of ovules in each carpel is a precise measure of the overall seed set. This method was also used by Jaeger and Després (1998), Després et al. (2007), and Lemke and Porembski (2012). Here, we counted the mean number of developing seeds in five undamaged carpels for each fruit, and divided it by the mean number of ovules for each population, calculated from a subset of 174 fruits (mean of 21.8 fruits per population, range: 5–68). This was possible as the variance in the number of ovules is low in all the populations studied, compared to the variance in the numbers of developing seeds (1.73 vs. 7.96) and allowed us to measure the seed set on a larger set of fruits. A previous study showed a positive correlation of fruit size with population size (Lemke and Porembski 2012). To control for this parameter in our analysis, we counted the number of carpels in each fruit.

To estimate the proportion of seeds released after predation, that is, the net seed set (Després et al. 2007; Ibanez et al. 2009), we calculated the proportion of seeds eaten, following the individual cost model developed by Després et al. (2007). The net seed set was calculated by multiplying seed set by 1 − 0.66*x* ^0.26^, where *x* was the egg density per carpel, calculated by counting the eggs and dividing by the number of carpels for each fruit (Lemke and Porembski 2012). This model was developed from empirical data gathered form populations spanning the wide geographic and ecological spectrum of *T. europaeus* and thus is a good approximation of the costs incurred by the plant in relation to the number of *Chiastocheta* larvae (Lemke and Porembski 2012).

### Self‐incompatibility test

*Trollius europaeus* was previously shown to be highly self‐incompatible by flower bagging experiments (Pellmyr 1989; Jaeger and Després 1998; Klank et al. 2010; Lemke and Porembski 2012). To assess the degree of self‐pollination in the studied populations, we subjected 15 flowers from different plants to the autonomous selfing experiment in each of the four largest populations (GRO‐2, KOB‐1, PEC‐2, and SIE‐3) in 2011. Flower buds were covered with dense nylon mesh to prevent pollinator visits and left untouched until dehiscence. Some flowers used for the study were lost during the experiment in population GRO‐2, so that the final number of collected fruits was 10 in this population. For each of the fruits, we counted the number of carpels and the total number of developed seeds. We calculated the proportion of seeds developing under self‐pollination treatment by dividing the number of seeds produced by the number of carpels and mean number of ovules in each population.

### Fitness‐related traits in *Trollius* populations

To assess fitness‐related traits of the progeny in the populations with and without *Chiastocheta*, we measured the size and germination rates of the seeds collected in 2011 in the four largest *T. europaeus* populations, GRO‐2, SIE‐3 (in which *Chiastocheta* were present), and KOB‐1, PEC‐2 (in which the flies were absent). We used only the largest populations to avoid the effect of small population sizes or unfavorable conditions on the seed set. In each population, we randomly collected 25 fruits, form separate plants, after dehiscence (29 in GRO‐2 population). For each fruit, as a proxy of seed size, we measured the mean mass of 25 undamaged, randomly sampled, and air‐dried seeds. Afterwards, the seeds were stored at −20°C and used for the germination experiment. After a pilot trial, the germination conditions were set as follows: 25 seeds were placed in a Petri dish on a filter paper soaked with 250 mg/mL solution of gibberellic acid (GA3) and stratified for 4 weeks in darkness at 4°C. Then, conditions were set to 20°C, 14:10 day:night light regime, with the position of the plates being randomized every 2 days. The number of germinated seeds was recorded after 2 weeks.

### Pollinator observations

In 2010 (pilot study) and 2011, we conducted series of observations in order to determine whether insects other than *Chiastocheta* flies visit flowers of *T. europaeus*. Each observation period consisted of 30 (in 2010) or 15 min (in 2011) of observation of 10 flowers, after which the observed group of flowers was changed. We conducted 63 observation periods (1035 min) for populations without (*n *=* *32 for KOB‐1, *n *=* *31 for PEC‐2) and 41 (615 min) for populations with *Chiastocheta* (*n *=* *16 for LUD‐2, *n *=* *3 for SIE‐2, *n *=* *9 for SIE‐3, *n *=* *13 for GRO‐2). All insects entering any of the observed flowers were determined at least to the family level and recorded. We calculated the visitation frequency as the number of visits per 15 min on 10 observed flowers.

In addition, to determine the presence as well as the relative abundance and the preference for *T. europaeus* flowers of each insect group, we checked the inside of flowers for the presence of insects in 2010 and 2011. We surveyed 561 flowers (*n *=* *210 for KOB‐1, *n *=* *207 for PEC‐2, *n *=* *36 for LUD‐2, *n *=* *50 for SIE‐3 and *n *=* *58 for GRO‐2). We did not include springtails (Collembola) and thrips (Thysanoptera) in the dataset.

### Data analysis

The statistical analysis was carried out in R (R Core Team 2014), version 3.1.2. The differences in the numbers of ovules in the studied populations were tested using one‐way analyses of variance (ANOVA).

For analyzing the seed set and net seed set data, we applied generalized linear mixed‐effects models (GLMM) using the lme4 package version 1.1.7 (Bates et al. 2014) with a binomial error family. We fitted populations and the interaction between the year and population in the random effect term, in order to account for non‐independence in the dataset caused by the repeated measures performed over 3 years. The fixed effects consisted in *Chiastocheta* presence, the fruit size (i.e., the number of carpels), and *log*‐transformed mean population size.

The effect of *Chiastocheta* presence on the seed germination, seed set, and net seed set, as well as the relationship between seed size and germination rate, in the four largest populations in 2011 was analyzed with binomial family GLMM using population identity as a random factor. We used only the four largest populations in order to reduce the effect of the population size on the seed set. We also calculated the relative fitness of the four populations, defined as a product of the mean net seed set and mean seed germination rate.

## Results

The size of the populations ranged from 5 to 17,300 flowers. A total of 647 fruits were used to determine the seed set, with an average of 47 per population × year combination (range: 4–174; see Table 1).

The mean fruit size ± standard deviation in the populations studied was 33.9 ± 11.8 carpels (range: 11–87). We did not find a significant relationship between the population size averaged over all years and fruit size (GLM: *z *=* *1.084, *P *=* *0.279). The mean number of ovules per carpel for all the populations was 10.8 ± 1.3 (*n *=* *174; range of mean values per population: 10.1–11.9). As the number of ovules varied significantly between populations (*F*_7,166_ = 4.49, *P *=* *0.001), we used the mean ovule number per carpel for each population to calculate the seed set. Because we calculated the proportion of seeds per mean number of ovules in populations, 2% (14) of the seed set values were larger than 1. For these fruits, we assumed the maximum seed set. Only 2% of the fruits (13) did not produce any seeds: 1% (3) for populations with and 3% (10) for populations without *Chiastocheta*.

Among the flowers subjected to the selfing treatment, 63.6% produced seeds, with a mean seed set of 0.02 ± 0.03 (range: 0–0.13, *n *=* *55). The seed set under selfing did not differ significantly between the studied populations (*F*_3,51_ = 0.32, *P *=* *0.811).

### Seed set and seed predation in relation to *Chiastocheta* presence

**Figure 2:**
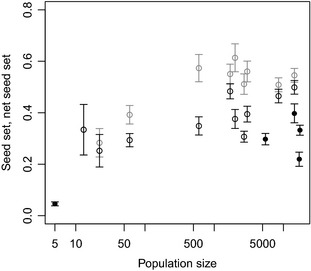
Relationship between *Trollius europaeus* population size and the seed set (in grey) and net seed set (in black) in populations hosting *Chiastocheta* (open points) and *Chiastocheta*‐free populations (closed points). Points represent mean ± standard errors. The population size is *log*‐transformed.

At the population level, the mean seed set was 0.41 ± 0.16 (*n *=* *15): 0.49 ± 0.11 (*n *=* *10) for populations with and 0.26 ± 0.14 (*n *=* *5) for populations without *Chiastocheta* (Fig. 2). Considering only the larger populations, that is, having more than 1000 flowers, the seed set was 0.45 ± 0.13 (*n *=* *10): 0.55 ± 0.04 (*n *=* *6) for populations with and 0.31 ± 0.07 (*n *=* *4) without the flies.

The net seed set was 0.37 ± 0.11 (*n *=* *15): 0.38 ± 0.08 (*n *=* *10) with and 0.25 ± 0.14 (*n *=* *5) without the flies. For larger populations, the average net seed set values were 0.38 ± 0.09 (*n *=* *10): 0.42 ± 0.07 (*n *=* *6), and 0.31 ± 0.07 (*n *=* *4), respectively.

Chiastocheta presence had a significant positive effect on the seed set and net seed set (*P* < 0.001 for both models; Table 2), as well as had the population size (*P* < 0.001 for both models). Fruit size had a significant effect only on the seed set (*P* = 0.006). The drop in seed set and net seed set values due to Chiastocheta absence was independent from site‐specific and site‐by‐year‐specific effects, which were included as random factors in both models.

**Table 2:** Effect of *Chiastocheta* presence, fruit size, and plant population size on the seed set, analyzed using binomial generalized linear mixed‐effects models (GLMM). Random effect terms control for the site‐specific and the site‐by‐year‐specific effects. The table shows estimates, stars denote the level of significance of *P*‐values, and standard errors are given in brackets

	Seed set	Net seed set
Intercept	−2.56 *** (0.37)	−2.44 *** (0.31)
*Chiastocheta* presence	1.19 *** (0.20)	0.68 *** (0.15)
Fruit size	0.01 ** (0.00)	0.00 (0.00)
Population size	0.16 *** (0.04)	0.16 *** (0.03)
*N* observations	647	647
*N* groups: Year:Population	15	15
*N* groups: Population	8	8
Variance: Year:Population.(Intercept)	0.05	0.03
Variance: Population.(Intercept)	0.01	0.00

***P < 0.001, **P < 0.01.

### Fitness‐related traits in *Trollius* populations

**Figure 3:**
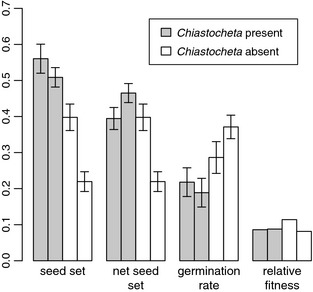
Seed size, net seed size, germination rate, and relative fitness (product of net seed size and germination rate) for the four largest *Trollius europaeus* populations in 2011. Bars represent the population mean ± standard errors.

Fitness‐related traits were studied in the four largest *T. europaeus* populations sampled in 2011. Estimated average net seed set was 0.41 ± 0.20 for populations with and 0.32 ± 0.23 for populations without *Chiastocheta*. The germination rate was 0.20 ± 0.21 and 0.33 ± 0.20, while the seed mass was 0.66 ± 0.14 and 0.91 ± 0.15 mg for the populations with and without the flies, respectively. Relative fitness was 0.09 for the two *Chiastocheta*‐free populations. The two *Chiastocheta*‐hosting populations had relative fitness of 0.11 and 0.08 (Fig. 3). We did not attempt to calculate confidence intervals for the relative fitness as the net seed set and germination rates were sampled from different individuals.

*Chiastocheta* presence had significant positive effect both on the seed set (*P *<* *0.001; Table 3; Fig. 3), and net seed set (*P *=* *0.048), but negative effect on the germination rate (*P *<* *0.001).

**Table 3:** Effect of *Chiastocheta* presence on the seed set, net seed set, and seed germination for the four largest populations in 2011. Data were analyzed using binomial generalized linear mixed‐effects models (GLMM). Population identity is fitted as a random effect. The table shows estimates, stars denote the level of significance of *P*‐values, and standard errors are given in brackets

	Seed set	Net seed set	Germination
Intercept	−0.85*** (0.21)	−0.85*** (0.20)	−0.72*** (0.11)
*Chiastocheta* presence	0.97*** (0.29)	0.55* (0.28)	−0.65*** (0.15)
*N* observations	195	195	104
*N* groups: Population	4	4	4
Variance: Population.(Intercept)	0.08	0.07	0.02

***P < 0.001, *P < 0.05.

### Insect observations in natural populations

**Figure 4:**
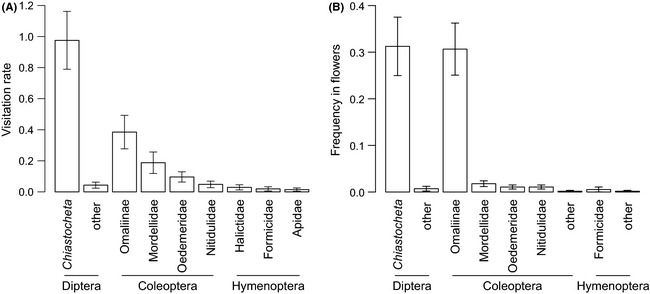
(A) Visitation rates of *Trollius europaeus* flowers by insects (number of visits per 10 flowers in 15 min). (B) Frequency of insects inside *T. europaeus* flowers (number of insects per flower).

In populations where they were present, *Chiastocheta* were the most frequent flower visitors (54% of visits), followed by beetles – Staphylinidae (Omaliinae, 21%), Mordellidae (10%, although mostly present in one studied population), and Oedemeridae (*Oedemera* genus, 0.7%). All Hymenoptera together were responsible for 4% of visits (Fig. 4A; see Table S1).

Insect groups found frequently inside *T. europaeus* flowers were *Chiastocheta* and small staphylinid beetles from the Omaliinae subfamily, both with a frequency of 0.31 insects × flower ^−1^. Other insect groups occurred with frequencies lower than 0.02 (Fig. 4B; see Table S1).

## Discussion

Nursery pollination systems have been used as a model for studying the evolution of mutualisms for decades (Janzen 1979). Among them, the interaction between *T. europaeus* and *Chiastocheta* flies has been the focus of a wide array of studies aiming at understanding the evolution of specificity in mutualisms (Jaeger et al. 2000, 2001; Després et al. 2007; Ibanez and Després 2009; Ibanez et al. 2009). Despite that, remarkably, the role of potential co‐pollinators and the assumption on the one‐to‐one nature of this and other systems have not been challenged and most of them are cited as examples of strictly obligate and specific mutualistic interactions between plants and pollinating seed parasites (Dufaÿ and Anstett 2003).

Indeed, it is claimed that *Chiastocheta* flies are the only pollinators of *T. europaeus* (Pellmyr 1989; Jaeger and Després 1998; Després et al. 2007). With this assumption, the analyses of costs and benefits for the plant suggested that the interaction is mutualistic over a wide geographic range (Pellmyr 1989; Després et al. 2007), as the plant produces more seeds than eaten by larvae and the numbers of released seeds remain stable despite variation in fly densities (Jaeger et al. 2001; Després et al. 2007). However, so far, no study could test the specificity hypothesis in a direct way from the plant perspective, due to the difficulty to set up an adequate experimental design, isolating the plants from *Chiastocheta*, while allowing the access of other insects.

Here, we take the opportunity of a natural experiment in southwestern Poland, where in some large but isolated *T. europaeus* populations, disturbance events led to a complete absence of flies. Therefore, we could directly measure the seed set and seed germination in the absence of a putative mutualistic partner. Moreover, by tracking part of the populations over consecutive years – in two of which the flies were present in the first, but missing in the next years, we could control for the site‐specific effects and disentangle it from the effects of *Chiastocheta* presence on the seed set.

### Is interaction with *Chiastocheta* obligate for the plant?

Contrasting with the previous studies (Pellmyr 1989; Jaeger and Després 1998; Després et al. 2007; but see Lemke and Porembski 2012 for preliminary results from 10‐fold smaller populations without *Chiastocheta*), we conclude that the interaction is not strictly obligate from the plant's perspective, as in the absence of *Chiastocheta* its relative female‐fitness does not decrease. This observation is of high importance, as analysis of costs and benefits for this system relied so far on the assumption that *Chiastocheta* are the only pollinators (Pellmyr 1989; Jaeger and Després 1998; Ferdy et al. 2002; Jaeger et al. 2001; Després et al. 2007; Lemke and Porembski 2012).

Although the seed set and net seed set in *Chiastocheta*‐free populations were on average 47% and 34% lower than in the populations with flies – thus confirming their important role as pollinators – the relative female‐fitness was equivalent between plant populations with and without flies.

Our results also confirm that self‐pollination cannot be considered as an alternative mechanism of seed production in *T. europaeus*, rather pointing to other flower visitors as pollen vectors (see below and Appendix S1). This corroborates results from other studies, also showing low or no selfing in populations throughout the range of *T. europaeus* (Pellmyr 1989; Jaeger and Després 1998; Klank et al. 2010; Lemke and Porembski 2012).

### Is fitness of **Trollius** europaeus reduced in the absence of Chiastocheta?

Our study not only shows that populations without *Chiastocheta* produce seeds, but also that their seeds are larger and germinate at a higher rate. Higher seed size is in line with the seed size‐number trade‐off theory (Smith and Fretwell 1974), where plant can allocate more resources per seed when the number of seeds is lower. The positive link between the seed size and germination within each of the studied populations confirms previous observations on other species, that larger seeds enhance seedling performance (e.g., Stanton 1984; Giles 1990; Simons and Johnston 2000; Sage et al. 2011). In addition, the link between seed size and fitness usually extends to the adult stage performance (Stanton 1984; Giles 1990; Baraloto et al. 2005).

We use a product of the net seed set and seed germination rate to compare the relative fitness of *Chiastocheta*‐free versus *Chiastocheta‐*hosting populations. Although we could not calculate confidence intervals for these estimates, the mean values of relative fitness for both types of populations were similar (Fig. 2), a result of the trade‐off between the number of initiated seeds and their germination rate. This shows that, at least in the studied populations, the fitness gain through the female‐flower‐function is disconnected from the presence *vs*. absence of *Chiastocheta*. We suggest that similar index should be used in the future for assessing the fitness gains in other pollination systems.

It is worth noting that at the population level, the net seed set was found to be independent of fly densities (Després et al. 2007) – populations with small fly densities produce less seeds but are less predated. In 36 European *T. europaeus* populations surveyed by Després et al. (2007), the mean net seed set was 0.46 ± 0.06, strikingly similar to the result from our larger *Chiastocheta*‐hosting populations. The effect we observe cannot be therefore accounted to lower fly densities in the studied populations. On the contrary, it should be even more pronounced when fly density is higher.

### Other insects as putative pollinators

Previous works on *T. europaeus* did not consider other flower visitors as pollinators, although the visitation rates by non‐*Chiastocheta* were described as being high in some areas (Ibanez et al. 2009). Contrary to Pellmyr (1989) who observed only bumblebees (*Bombus* spp.) entering the flowers, Jaeger and Després (1998) observed a range of other flower visitors from the Hymenoptera (*Bombus* spp., Vespidae), Coleoptera, and Diptera (Syrphidae) orders, representing 9% of total flower visitors. Ibanez et al. (2009) observed other Diptera (Syrphidae), Coleoptera, Hymenoptera (mostly Apidae), and Hemiptera accounting for 28% of visits in low elevations, with Syrphidae being the most frequent visitors. At subalpine elevations, other visitors were much less common, with only Diptera (mostly Syrphidae) and Hymenoptera (Tenthredinidae), contributing to around 2% of the visits.

**Figure 5:**
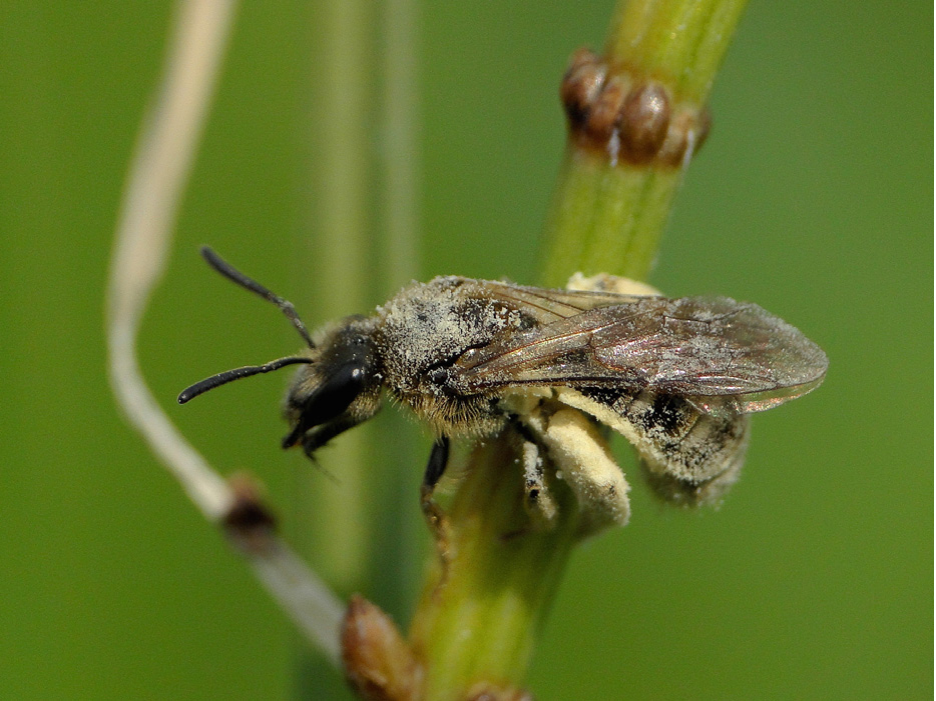
Small *Halictidae* bee carrying *Trollius europaeus* pollen after visiting the flower.

In the populations included in this study, the only insect groups that had high visitation rates were beetles: Staphylinidae (Omaliinae), Mordellidae, and Oedemeridae (genus *Oedemera*) (Fig. 4A; see Table S1), although only Omaliinae were frequently found inside the flowers (Fig. 4B; see Table S1). Last but not least, we observed multiple evidences of those beetles, as well as representatives of all insect groups mentioned above, carrying *T. europaeus* pollen (Fig. 5; T. Suchan, unpubl. obs.). Nevertheless, additional observations, including counting pollen grains from insects’ bodies, are necessary to assess their relative importance as pollinators (see also Appendix S1 for the detailed discussion).

### Asymmetric nature of *Trollius*–*Chiastocheta* interaction

While flies require the plant to reproduce, the plant can produce seeds without the flies. Such asymmetry is suggested to be a common feature of plant–pollinator interactions (Bascompte et al. 2003; Vázquez and Aizen 2004), where specialized pollinators tend to interact with more generalist plants and *vice versa*. This also applies to *T. europaeus*, which is more generalist than suggested by previous studies (Pellmyr 1989; Jaeger and Després 1998; Després et al. 2007; Klank et al. 2010; Lemke and Porembski 2012).

One should keep in mind, however, that in harsh conditions where non‐*Chiastocheta* pollinators might be scarce, the pollination system might tend to be more symmetrical. For instance, Ibanez et al. (2009) found that the availability of alternative pollinators varied with elevation. The interaction can thus be variable across climatic dimensions, being more symmetrical in high mountain habitats where alternative pollinators are rare. This also points to the possible evolutionary origin of the interaction, which could evolve in the scarcity or low effectiveness of other pollinators (Ibanez and Després 2009).

The variation in costs and benefits in nursery pollination systems according to the availability of co‐pollinators has already been shown to vary in the case of *Lithophragma* (Thompson and Pellmyr 1992; Thompson and Fernandez 2006) and *Silene* (Pettersson 1991, 1992), but is demonstrated for the first time in the *T. europaeus*–*Chiastocheta* interaction, previously considered as a strict mutualism. These observations put some previous data on this system in a different perspective, highlighting the role of *Chiastocheta* as seed parasites and the role of community context in defining interaction's outcomes.

### Reconsidering traits affecting stability of nursery pollination systems

Presence of co‐pollinators in nursery pollination systems is intriguing, because it poses the question why the plant does not evolve out of the interaction, toward use of alternative, nonparasitic pollinators (Holand and Fleming 2002).

From the female flower function perspective, the seed set in *Chiastocheta*‐free populations is still lower than that released after predation in *Chiastocheta*‐hosting populations. However, the seed germination rate–seed set trade‐off can outbalance any positive effects of *Chiastocheta* presence. In our populations, the flies do not act as mutualists, as the plant does not demonstrate any fitness gain in terms of the number of offspring. One would therefore expect that in the presence of other pollinators, the plant should be under selection to reduce seed predation. Despite that, the interaction between *T. europaeus* and *Chiastocheta* seems stable over its geographic range (Després et al. 2007; Espíndola et al. 2014).

We hypothesize that this apparent paradox can be explained by maximization of by‐product benefits (*sensu* Connor 1995) supplied by the flies through the male flower function. As *Chiastocheta* strongly favor closed floral morphology of *T. europaeus*, their visits lead to significantly higher pollen export from the closed flowers (Ibanez et al. 2009). By being the most efficient pollen dispersers (Ibanez et al. 2009), *Chiastocheta* can exert selective pressure for preferred floral morphology (Stebbins 1970). Thus the closed, globelike flower morphology is selected not to exclude other flower visitors, but by the innate preference of *Chiastocheta* flies. Closed morphology is thus maintained even if seed production alone could be higher in the absence of the parasite. This highlights not only the importance of the conflict between insect reproduction and the number of seeds produced, but also between the male and female flower functions (Lankinen and Larsson 2009) in nursery pollination systems.

The model proposed here, which may be applied to the evolution of other nursery pollination systems, is thus different from the ones suggested earlier, where both partners benefit from the interaction and coevolve through maximization of reciprocal benefits in a tit‐for‐tat model of coevolution (Axelrod and Hamilton 1981). Instead, it depicts a scenario where an originally antagonistic interaction brings some by‐product benefits for the plant (Connor 1995), in the form of more efficient pollen dispersal, and is later fixed by the fly preference toward the closed flower morphology, by excluding or reducing the visitation rates of other visitors. Therefore, it might be interpreted as a form of evolutionary compensation, leading to a mutual codependence between partners (Aanen and Hoekstra 2007). It thus makes the case against analyzing such interactions in a strict coevolutionary framework (Suchan and Alvarez 2015); and argues in favor of underlining the role of conflict and suboptimal local adaptive peaks in its evolution.

Finally, we argue that focusing on the benefit to cost ratio in such interactions, incorporating the presence of alternative pollinators and seed production–seed germination trade‐off can provide new and exciting avenues of research.

## Conflict of Interest

The authors declare that they have no conflict of interests and that experiments comply with the current laws of Poland, where they were performed.

## Data accessibility

Data available from the Dryad Digital Repository (http://doi.org/10.5061/dryad.640qp).

## Supporting information


**Appendix S1.** Putative co‐pollinators of *Trollius europaeus*.

**Table S1.** Visitation rates and frequency of insects found in *Trollius europaeus* flowers.
