# Supplementary material for: Asymmetrical nature of the Trollius–Chiastocheta interaction: insights into the evolution of nursery pollination systems
Source: Ecol Evol. 2015 Oct 8;5(21):4766–77. doi: 10.1002/ece3.1544 (PMC4662325; doi:10.1002/ece3.1544)
Supplement: Supplementary file 1 — Appendix S1. Putative co‐pollinators of Trollius europaeus. [file ECE3-5-4766-s001.docx]

**Appendix S1** Putative co-pollinators of *Trollius* *europaeus*.

In the studied populations, the only insect groups that had high visitation rates were Staphylinidae (Omaliinae), Mordelliade and Oedemeridae (genus *Oedemera*) beetles (ESM 2).

Whereas Mordellidae were only reported in abundance from one single population — and no other observations are depicted in the literature — Staphylinidae and Oedemeridae were already reported from Alpine populations. Small Omaliinae have not been considered as effective pollinators so far because of their low pollen carrying capacity and visitation rates (Lindsey 1984; Escaravage & Wagner 2004), significantly contributing to pollination only in rare cases (Zamora 1999). Their role in pollen transfer however cannot be excluded and pollen grains on visitor’s bodies (T. Suchan, pers. obs.) should be counted to confirm the role of this group as a pollinator. While, because of their small body size, the amount of pollen transferred is probably low, their high population densities and relatively high visitation rates make them a putative pollen vector in the studied populations. Oedemeridae, albeit having lower frequencies and visitation rates may possibly transfer more pollen given their larger size.

In contrast to previous observations (Jaeger & Després 1998; Ibanez et al. 2009), we did not observe Syrphidae in the studied populations, whereas we could observe large numbers of them in middle- to high-elevation populations in the Alps, Jura and Tatra Mountains (T. Suchan, unpublished data). Also, whereas not recorded in the studied populations, *Bombus* spp. were shown to visit flowers in populations from Scandinavia (Pellmyr 1989), the Alps (Ibanez et al. 2009) and the Sudety Mountains (T. Suchan, unpublished data). In the latter area, we found evidence that they are active pollen vectors, even among patches of plants located 50 m apart (Suppl. Fig. 1). This points to the conclusion that the species composition of alternative pollinators might be geographically variable and that this might lead to contrasting local adaptation from the plant’s perspective.


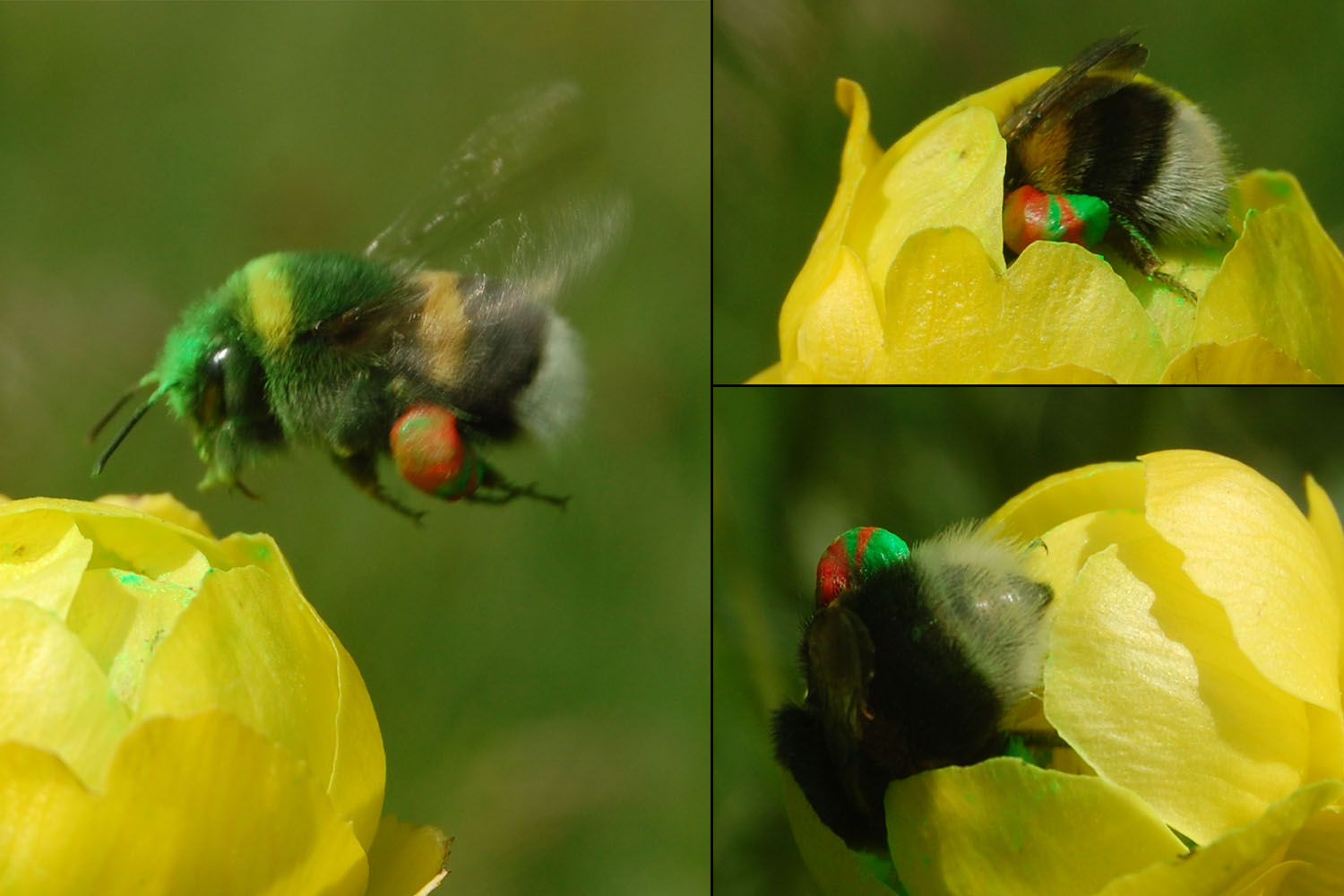


*Fig. 1*. *Bombus* sp. individual carrying fluorescent dye collected several times between two groups of *Trollius europaeus* plants, with their anthers marked with green or orange dye respectively. The two groups of plants were 50 m apart (T. Suchan, unpublished data). Note that the individual made at least two recursive visits to each of the groups of plants and that a significant amount of dye is carried on the insect’s body.

**References**

Lindsey, A.H. (1984) Reproductive biology of Apiacea. I. Floral visitors to *Thaspium* and *Zizia* and their importance in pollination. American Journal of Botany 71, 375–387. doi: [10.2307/2443496](http://dx.doi.org/10.2307/2443496)

Escaravage, N. & Wagner, J. (2004) Pollination effectiveness and pollen dispersal in a *Rhododendron ferrugineum* (Ericaceae) population. Plant Biology 6, 606–15. doi: [10.1055/s-2004-821143](http://dx.doi.org/10.1055/s-2004-821143)

Zamora, R. (1999) Conditional outcomes of interactions: the pollinator-prey conflict of an insectivorous plant. Ecology 80, 786–795. doi: 10.1890/0012-9658(1999)080[0786:COOITP]2.0.CO;2

Jaeger, N. & Després, L. (1998) Obligate mutualism between *Trollius europaeus* and its seed-parasite pollinators *Chiastocheta* flies in the Alps. Comptes Rendus de l’Académie des Sciences - Series III - Sciences de la Vie 321, 789–796. doi: [10.1016/S0764-4469(98)80019-X](http://dx.doi.org/10.1016/S0764-4469(98)80019-X)

Ibanez, S., Després, L. & Dujardin, G. (2009) Stability of floral specialization in *Trollius europaeus* in contrasting ecological environments. Journal of Evolutionary Biology 22, 1183–1192. doi: [10.1111/j.1420-9101.2009.01731.x](http://dx.doi.org/10.1111/j.1420-9101.2009.01731.x)

Pellmyr, O. (1989) The cost of mutualism: interactions between *Trollius europaeus* and its pollinating parasites. Oecologia 78, 53–59. doi: [10.1007/BF00377197](http://dx.doi.org/10.1007/BF00377197)
