# Supplementary material for: Asymmetrical nature of the Trollius–Chiastocheta interaction: insights into the evolution of nursery pollination systems
Source: Ecol Evol. 2015 Oct 8;5(21):4766–77. doi: 10.1002/ece3.1544 (PMC4662325; doi:10.1002/ece3.1544)
Supplement: Supplementary file 2 — Table S1. Visitation rates and frequency of insects found in Trollius europaeus flowers. [file ECE3-5-4766-s002.docx]

**Table S1** Visitation rates and frequency of insects found in *Trollius europaeus* flowers .

|  | Visitation rate | | | Frequency | | |
| --- | --- | --- | --- | --- | --- | --- |
| Species | mean | sd | *n* | mean | sd | *n* |
| Chiastocheta | 0.98 | 1.19 | 41 | 0.31 | 0.75 | 144 |
| other Diptera | 0.04 | 0.20 | 104 | 0.01 | 0.12 | 561 |
| Omaliinae | 0.38 | 1.10 | 104 | 0.31 | 1.33 | 561 |
| Mordellidae | 0.19 | 0.70 | 104 | 0.02 | 0.15 | 561 |
| Oedemeridae | 0.10 | 0.33 | 104 | 0.01 | 0.10 | 561 |
| Nitidulidae | 0.05 | 0.21 | 104 | 0.01 | 0.10 | 561 |
| other Coleoptera | 0.03 | 0.17 | 104 | 0.00 | 0.04 | 561 |
| Apidae | 0.01 | 0.11 | 104 | 0.00 | 0.00 | 561 |
| Formicidae | 0.02 | 0.14 | 104 | 0.01 | 0.13 | 561 |
| other Hymenoptera | 0.00 | 0.00 | 104 | 0.002 | 0.04 | 561 |

Visitation rate – visits per 10 flowers in 15 minutes.

Frequency – number of insects found per flower.
